# Ultrasound-assisted deep eutectic solvent of neuroprotective extracts from *Paeonia lactiflora* Pall. root: Process optimization, compositions characterization, and neuroprotective activity validation

**DOI:** 10.1016/j.ultsonch.2025.107449

**Published:** 2025-06-27

**Authors:** Yongkang Xue, Zhengyu Hu, Yuxin Jiang, Man Li, Yanan Liu, Yuanqi Duan, Wei Zhou, Jinfeng Sun, Gao Li

**Affiliations:** Key Laboratory of Natural Medicines of the Changbai Mountain, Ministry of Education, College of Pharmacy, Yanbian University, Yanji 133002 Jilin Province, China

**Keywords:** *Paeonia lactiflora* Pall. root, Ultrasound-assisted extraction, Deep eutectic solvent

## Abstract

The objective of this study was to efficiently extract components with neuroprotective activity from the root of *Paeonia lactiflora* Pall. (RPL). The neuroprotective extracts of RPL were extracted by ultrasound-assisted deep eutectic solvents (DESs) method, and optimized by response surface methodology with PC12 cell viability as a guide. The optimal process: DESs (composed of L-Proline and Glycerol, Betaine monohydrate and Benzyl alcohol in a ratio of 7:3 (v/v) with 60 % water), extraction time 22 min, liquid-to-solid ratio 42 mL/g, temperature 51 °C, ultrasonic power 243 W. These conditions yielded extracts with cell viability 81.44 ± 0.18 % (n = 3), which is much higher than conventional heat and standard DESs extraction methods. Then, the RPL neuroprotective purified extract (RNPE) was obtained by AB-8 macroporous resin, and characterized by UPLC-Q-TOF-MS analysis. The results showed that RNPE was mainly composed of 50 compounds, including terpenoids, phenolics, sterols and so on, and it showed significant neuroprotection in middle cerebral artery occlusion (MCAO) mice. These results established ultrasound-assisted deep eutectic solvent extraction (UADE) as a sustainable approach for extracting neuroprotective agents from RPL, combining process efficiency with bioactive preservation. The integrated method, from screening of DESs to optimization of ultrasound-assisted extraction conditions and *in vivo* validation, establishes an efficient template for neuroprotective components.

## 1 Introduction

Stroke, the second highest contributor of global mortality, affects approximately 7.25 million individuals annually, with profound public health impacts due to its significant contribution to disability [[1], [2], [3]]. Ischemic stroke (IS), representing over 62 % of all stroke incidents, is a vascular central nervous system disease resulting from cerebral artery occlusion caused by thrombosis [4]. The primary interventions for IS remains intravenous thrombolysis and mechanical endovascular thrombectomy, which involve ischemic area blood circulation restore [5,6]. However, these methods face constraints due to a restricted treatment timeframe and unavoidable cerebral ischemia–reperfusion injury (CIRI) that follows, with only a very small proportion of patients benefit from this technique [7,8]. Combining neuroprotection with intravenous or mechanical endovascular thrombectomy is now a crucial next step for advancing CIRI treatment [9]. Recently, natural products demonstrate significant potential in neuroprotection with the technological development of natural products-based drug discovery [10]. Natural products are addressing these challenges and opening up new opportunities in CIRI.

*Paeonia lactiflora* Pall. (*P. lactiflora*), an herbaceous perennial indigenous to temperate Asia, with significant ethnobotanical applications spanning traditional medicine, horticulture, and culinary [11]. The root of *P. lactiflora* (RPL) has been employed in traditional medicine for millennia, primarily for its heat-clearing, blood-cooling, and blood stasis-resolving properties [12]. And recent studies have demonstrated its significant neuroprotective effects, including antidepressant, sedative, analgesic, and anticonvulsant activities [[13], [14], [15]]. RPL have been shown to contain abundant bioactive compounds, including: monoterpene glycosides, diterpenoids, flavonoids and phenolics, which contribute to RPL’s observed neuroprotection, particularly in alleviating nerve injury [12,[16], [17], [18], [19], [20]]. However, conventional extraction of bioactive compounds from RPL primarily relies on maceration, percolation, and solvent extraction methods, which are time-consuming, solvent-intensive, cost-ineffective, and lack selectivity [11,[20], [21], [22]]. Therefore, enhancing the extraction efficiency of bioactive compounds from RPL represents a crucial research challenge.

Deep eutectic solvents (DESs) have recently gained recognition as sustainable alternatives for natural product extraction. Consisting of hydrogen bond donors and acceptors, DESs systems exhibit advantageous properties including low toxicity, high biodegradability, tunable physicochemical characteristics, and bioactivity-enhancing effects, establishing them as exemplary green extraction media [[23], [24], [25], [26]]. Additionally, ultrasound-assisted extraction (UAE) synergistically enhances DESs-based extraction through leveraging cavitation, micro jetting, and mechanical vibrations to disrupt plant cell walls, improve solvent penetration and mass transfer [27,28]. The synergistic combination of DESs and UAE demonstrates significant potential for the efficient extraction of medicinal plant constituents, while effectively maintaining their structural integrity and biological activity [[29], [30], [31]]. Therefore, ultrasound-assisted deep eutectic solvent extraction (UADE) represents an effective strategy for extracting bioactive compounds from RPL.

This study aims to extract neuroprotective compounds from RPL using UADE. The extraction process was optimized with bioactivity-guided approach, followed by comprehensive *in vivo* validation and phytochemical characterization of extracts. The findings are intended to contribute to the utilization of RPL and the broader application of UADE.

## 2 Materials and methods

### 2.1 Materials and reagents

The RPL material was sourced from Bozhou Chinese herb market in Anhui, China. L-Proline (Pro), betaine monohydrate (BM), glycerol (Gly), benzyl alcohol (BA) and edaravone (Eda) were all purchased from Aladdin (Shanghai, China) with over 98 % purity. Reagents for cell culture, including Dulbecco’s modified Eagle medium (DMEM) (L100 and L1021), and Fetal bovine Serum (FBS) (F803), were obtained from Baidi biotech (Zhejiang, China). The cell counting kit-8 (CCK-8) was purchased from Apexbio (Houston, USA). 2, 3, 5-triphenyltetrazolium chloride (TTC) were acquired from Sigma-Aldrich (Shanghai, China). All other chemicals and reagents used were HPLC or analytical grade, employed as received.

### 2.2 Preparation of DESs

All DESs were synthesized using established methods [23,32]. In brief, predetermined molar ratios of DESs components (Table S1) were precisely mixed, and heated at 80 °C under continuous stirring until achieving homogeneity. Additionally, two-phase DESs were developed with hydrophilic deep eutectic solvent (PDES), hydrophobic deep eutectic solvent (HDES) (0:10–10:0 ratios) and water (0–90 % content). The functional groups of the DESs were evaluated using a Fourier transform infrared spectrometer (FTIR) (Nicolet iS20, Thermo Fisher Scientific Inc., Waltham, MA, USA) within the wavelength range of 400–4000 cm^−1^.

### 2.3 Ultrasound-assisted DESs extraction

RPL samples were pulverized using a tube mill and sieved (60-mesh) to obtain uniform powder. The UADE procedure was performed at 50 °C and 200 W using an ultrasonic bath (JM-15D-28, Guangdong Jiemeng Ultrasonic Technology Co. Ltd., Shenzhen, China). Briefly, 100 mg RPL powder was extracted with 3 mL of DESs using ultrasound assistance for 40 min. Following cooling, the mixture underwent centrifugation (10,000 rpm, 30 min) to isolate the RPL extract supernatant.

### 2.4 Activity-guided optimization of UADE method

#### 2.4.1 Screening of the optimal DESs

Using the various DESs prepared in Section 2.2, neuroprotective extracts from RPL were obtained following the extraction protocol detailed in Section 2.3. The extracts were then evaluated for neuroprotective activity using an OGD/R model in PC12 cells as a bioactivity screening platform. In brief, the OGD/R model was established in PC12 cells as an *in vitro* simulation of CIRI. PC12 cells (Baidi Biotech, Zhejiang, China) were maintained in DMEM supplemented with 10 % FBS and 1 % penicillin–streptomycin (100 IU/mL-100 μg/mL) at 37 °C with 5 % CO_2_. Following 2 h pretreatment with different RPL extracts, cells were underwent OGD/R induction. The OGD/R model was created by maintaining PC12 cells in glucose/serum-deprived DMEM under controlled hypoxia (1 % O_2_, 5 % CO_2_ and 94 % N_2_) for 10 h at 37 °C. After hypoxia, the cells were returned to normoxia (5 % CO_2_, 95 % air) and reoxygenated in high-glucose DMEM for 24 h [33,34]. The cell viability was detected by the general CCK-8 method and calculated using the equation (Eq) 1.

(1) $$Relative\;cell\;viability\;\left(\%\right)\;=\;\left(A_{experiment}-A_{blank}\right)/\left(A_{control}-A_{blank}\right)\;\times \;100\%$$

#### 2.4.2 Single-factor experiment

The factors influencing RPL extraction were investigated. The univariate factors encompassed the liquid-to-solid ratio (10, 20, 30, 40, and 50 mL/g), temperature (30, 40, 50, 60, 70, and 80 °C), ultrasonic power (50, 100, 150, 200, 250 and 300 W) and extraction time (10, 20, 30, 40 and 50 min). Each parameter was investigated while maintaining other variables constant (30 mL/g, 50 °C, 200 W, and 40 min) to isolate individual effects on neuroprotective components extraction.

#### 2.4.3 Response surface methodology (RSM)

Building on single-factor optimization results, four factors: extraction time (X_1_), liquid to material ratio (X_2_), temperature (X_3_) and ultrasonic power (X_4_), were optimized using RSM. The cell viability of the extract was regarded as the primary response (Y). A three-level, four-factor Box-Behnken Design (BBD) was implemented via Design-Expert software (13.0.5.0) to model and predict optimal extraction conditions (Table S2). The BBD comprised 29 experimental runs, including 24 factor experiments and 5 central experiments for error estimation. Independent variable levels (coded and actual) are shown in Table 1. Response data were fitted to a quadratic model:

(2) $$Y=\alpha_{o}+\sum_{i=1}^{k}{\alpha_{i}X}_{i}+\sum_{i=1}^{k}\alpha_{\mathrm{ii}}X_{i}^{2}+\sum_{i=1}^{k}\sum_{j=i+1}^{k-1}\alpha_{\mathrm{ij}}X_{i}X_{j}$$

**Table 1:** BBD experiments and results.

Run	X_1_, (min)	X_2_, (mL/g)	X_3_, (°C)	X_4_, (W)	Y, (%)
1	10	30	50	250	78.43
2	30	30	50	250	77.97
3	10	50	50	250	78.79
4	30	50	50	250	80.07
5	20	40	40	200	77.92
6	20	40	60	200	78.90
7	20	40	40	300	78.19
8	20	40	60	300	78.32
9	10	40	50	200	78.42
10	30	40	50	200	79.84
11	10	40	50	300	78.16
12	30	40	50	300	78.75
13	20	30	40	250	78.24
14	20	50	40	250	78.33
15	20	30	60	250	78.69
16	20	50	60	250	78.87
17	10	40	40	250	78.07
18	30	40	40	250	78.21
19	10	40	60	250	78.41
20	30	40	60	250	78.92
21	20	30	50	200	79.55
22	20	50	50	200	79.75
23	20	30	50	300	78.17
24	20	50	50	300	79.09
25	20	40	50	250	81.07
26	20	40	50	250	81.21
27	20	40	50	250	81.76
28	20	40	50	250	81.38
29	20	40	50	250	81.12

### 2.5 Model validation and comparison of extraction methods

Model reliability was confirmed through experimental verification using the BBD-derived optimal parameters. Moreover, the heat extraction was performed using optimized UADE parameters (extraction time at 22 min, liquid-to-solid ratio at 42 mL/g, temperature at 51 °C, ultrasonic power at 243 W) using four individual solvents in separate extraction processes: distilled water extraction (H_2_O-E), ethanol (EtOH-E) extraction, methanol extraction (MeOH-E) and DESs extraction (DESs-E). While ultrasound-assisted distilled water extraction (UA-H_2_O-E) was conducted to assess the relative performance of the UADE. The neuroprotective activity evaluation was assessed following Section 2.3 protocols. Extraction yield was calculated by the weights of initial RPL material and extracted residue after solvent removal.

### 2.6 Purification of neuroprotective components

The RPL extracts were prepared using optimized UADE parameter (Section 2.3), and subsequently purified using AB-8 macroporous resin [35,36]. Briefly, the RPL extracts were chromatographed on an AB-8 resin (1.6 × 50 cm), eluted with ethanol (1.5 BV/h, 4 BV). And the eluate was collected, concentrated via rotary evaporation, and vacuum-dried at 55 °C to yield the RPL neuroprotective purified extract (RNPE).

### 2.7 Characterization of compositions by UPLC-Q-TOF-MS

UPLC-Q-TOF-MS analysis was conducted using ACQUITY UPLC H-Class PLUS system in conjunction with the Xevo G2-XS QTOF mass spectrometer (Waters, MA, USA). The chromatography separation was achieved on ACQUITY UPLC BEH C18 column (2.1 × 50 mm, 1.7 μm). The mobile phases employed were 0.1 % formic acid in water (phase A) and a mixture of 0.1 % formic acid in acetonitrile (phase B). A constant flow rate of 0.4 mL/min was maintained, and the column temperature was regulated at 40 °C throughout the analysis. The column temperature was set at 40 °C. A linear gradient elution was performed as detailed in Table S3. The mass spectrometer featured electrospray ionization (ESI) capability in positive and negative ion modes. The mass spectrometry parameters were configured as follows: the scan range for *m*/*z* was set between 50 and 1200, the ESI source temperature was maintained at 110 °C, the spray voltage was 1500 V (positive ions) and 2000 V (negative ions), and the cone voltage ranged from 20 to 40 V.

### 2.8 Neuroprotective of RNPE in the MCAO model

#### 2.8.1 Animals

Male C57BL/6 mice (8-week-old) were sourced from and maintained at Experimental Animal Center of Yanbian University. Mice were maintained under controlled conditions (23 ± 2°C, 55 ± 5 % humidity, 12 h light/dark cycle) with free access to food and water. All procedures were approved by the Animal Ethics Committee of Yanbian University (YD20240311004) and compliant with the National Institutes of Health Guide for the Care and Use of Laboratory Animals.

#### 2.8.2 MCAO model establishment

Following isoflurane anesthesia, mice underwent MCAO was induced via ventral midline neck incision with careful separation and ligation of the common carotid, external carotid, and pterygopalatine arteries. Microsurgery was then used to perform a small cut on the external carotid artery. A filament was inserted through the incision to induce ischemia for 1.5 h with subsequent 24 h reperfusion after filament removal [37]. The control group received identical procedures excluding filament insertion [38]. 35 mice were randomized into five experimental groups (n = 7 per group): (1) Sham; (2) MCAO; (3) MCAO + 10 mg/kg RNPE; (4) MCAO + 20 mg/kg RNPE; (5) MCAO + 10 mg/kg Eda. The treatment was administered via intraperitoneal injection (once daily × 7 days) before MCAO induction. Sham and MCAO controls received isovolumetric saline [39].

#### 2.8.3 mNSS test

Neurological function was assessed at 24 h reperfusion using the modified neurological severity score (mNSS) test [40,41]. The mNSS evaluates motor, sensory, reflex, and balance functions (score range: 0–14; higher scores indicate greater impairment).

#### 2.8.4 Gait trajectory testing

Gait trajectory analysis can further assess the degree of neurological functional impairment. Specifically, ink was used to record the footprints of the mice after reperfusion for 24 h.

#### 2.8.5 TTC staining

Following anesthetized euthanized mice, the brain tissues were harvested and sectioned coronally. The sections were placed in 1 % TTC (37 °C, 30 min, dark incubation) with periodic inversion to ensure uniform staining. Subsequently, stained brain sections were photographed for analysis. Infarct volume percentage was calculated by analyzing five consecutive brain sections using ImageJ software and normalized to total brain volume.

### 2.9 Statistical analysis

The data were presented as mean ± standard deviations (SD). Group differences were assessed by one-way an analysis of variance (ANOVA) with multiple comparisons, conducted in GraphPad Prism 8.0 (significance: *p* < 0.05).

## 3 Results and discussion

### 3.1 Screening of DESs

**Fig. 1:**
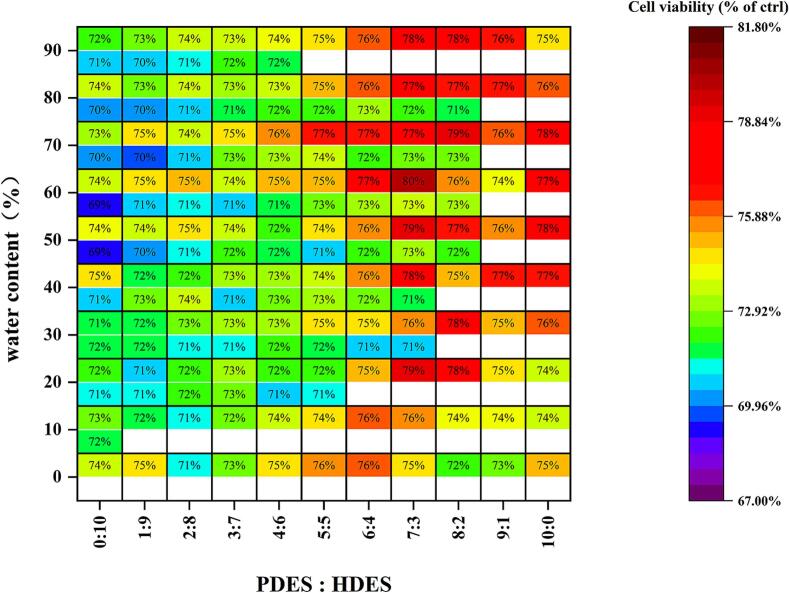
The cell viability assessment of 110 DESs and their corresponding upper/lower phase extracts (white line indicates phase separation).

To extract neuroprotective constituents from RPL, the UADE method was employed. Initially, a PDES was prepared by mixing L-proline and glycerol, and a HDES was synthesized by combining betaine and benzyl alcohol. Subsequently, 110 DESs were systematically developed, and the effects of varying PDES-to-HDES ratios, as well as deionized water content, on the neuroprotective activity of RPL extracts were evaluated to optimize the extraction process. As illustrated in the Fig. 1, 64 DESs formed a biphasic system. Notably, upper-phase extracts demonstrated greater neuroprotection than lower-phase extracts, as determined by the cell viability. Consequently, we prioritized the upper-phase extracts for further analysis, along with single-phase solvent extracts. Extracts obtained from PDES:HDES ratios of 6:4 to 10:0 demonstrated significantly greater neuroprotective activity compared to ratios of 0:10 to 5:5. This observed polarity dependent activity gradient suggests preferential solubility of the bioactive neuroprotective components in solvent systems with higher polarity [18,20]. Consistent with established dissolution theory, increasing solvent polarity through hydrophilic solvent addition enhanced the dissolution and extraction efficiency of bioactive components. Intriguingly, while water incorporation improved extract activity, no clear dose-dependent relationship was observed. This phenomenon may be result from aqueous modulation of DESs rheological properties, decreasing viscosity, which facilitates the extraction process. Additionally, water induced alterations of the hydrogen bonding and electrostatic balance within the DESs, thereby decreasing molecular interactions between the solvent and bioactive compounds [42,43]. Ultimately, the optimal DESs extraction system was identified as a PDES:HDES ratio of 7:3 containing 60 % water (upper-phase), yielding extracts that demonstrated 79.86 ± 0.88 % cell viability.

Additionally, the structure of the optimal DESs was confirmed by FTIR as shown in Fig. S1. In the FTIR spectrum of Gly, two characteristic bands appear at 3397 cm^−1^ and 1042 cm^−1^, corresponding to the O–H stretching and bending vibrations, respectively. The absorption band at 1638 cm^−1^ was unambiguously assigned to the C=O stretching vibration [44]. In contrast, the spectrum of Pro displayed characteristic O–H bond absorption at 3427 cm^−1^ and N–H bond bending vibration at 1407 cm^−1^. The characteristic peak of the C=O stretching vibration was observed at 1622 cm^−1^ [45]. Upon analyzing the FTIR spectrum of the synthesized PDES, a notable shift in the O–H bond absorption to 3386 cm^−1^ is observed, along with characteristic absorptions from both Gly and Pro. This shift suggests the formation of PDES through intermolecular hydrogen bonding. Similar structural characteristics were demonstrated for HDES [46,47]. Further investigation in the FTIR spectrum of the optimal DESs revealed significant shifts in characteristic absorption bands: the O–H stretching vibration shifted to 3346 cm^−1^ and the bending vibration to 1022 cm^−1^. Notably, the spectra maintained characteristic absorptions corresponding to both PDES and HDES [42,48]. These spectral modifications provide clear evidence for the formation of the optimal DESs through intermolecular hydrogen bonding between the constituent components.

### 3.2 Single factor experiments

#### 3.2.1 Effect of temperature on neuroprotective activity of extracts

**Fig. 2:**
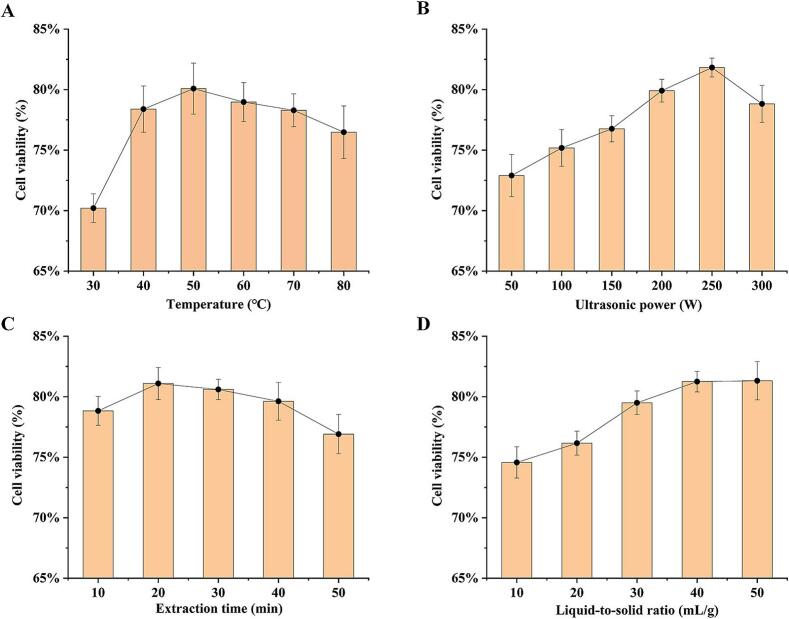
Effects of factors on neuroprotective activity of RPL extracts. (A) temperature; (B) ultrasonic power; (C) extrcation time; (D) liquid-to-solid ratio.

Temperature significantly influenced the neuroprotective efficacy of the extracts. As illustrated in Fig. 2A, the neuroprotective activity exhibited temperature-dependent response, with a significant positive correlation observed between 30 °C and 50 °C. This trend may be attributed to the diminished intermolecular interactions among chemical components and the intensification of molecular motion as the temperature increases. These changes enhance the binding affinity between active components and the solvent, which enables compound solubility, ultimately augmenting neuroprotective activity [35]. However, excessively high temperatures can degrade bioactive compounds and negatively impacting the activity of the extracts [44]. This consistent trend has also been documented in the extraction of polyphenols from pomegranate seeds and phyllanthi fructus, as well as anthocyanins from *Vitis davidii* Foex. Pomace [[49], [50], [51]]. Therefore, the optimal extraction temperature has been identified as 50 °C, achieving a balance between extraction efficiency and compound stability. These results highlight the critical need for precise temperature control in UADE to maximize the extracts activity while preventing potential thermal decomposition.

#### 3.2.2 Effect of ultrasonic power on neuroprotective activity of extracts

The influence of ultrasonic power on the neuroprotective efficacy of the extracts was also examined, as it represents a critical parameter of extraction. The results demonstrated that the activity of the extracts initially increased but subsequently decreased with higher ultrasonic power, as illustrated in Fig. 2B. The extracts demonstrated peak neuroprotective activity of 81.82 ± 0.78 % cell viability at an ultrasonic power of 250 W. This enhancement primarily results from the cavitation effects induced by ultrasound. Cavitation-induced microjets disrupt cellular matrices, enhancing intracellular component release. Additionally, the accompanying mechanical effects enhance solvent diffusion and improve the contact between the active components and solvent, thereby increasing the mass transfer rates [52]. However, excessive ultrasonic power promotes ineffective bubble formation, increasing acoustic scattering and diminishing cavitation efficiency [53]. In addition, excessively high ultrasonic power can induce structural alterations in active components, leading to a reduction in the activity of the extracts [54]. A phenomenon akin to that also observed in the extraction of flavonoids from *Ginkgo biloba* and sea buckthorn fruits [45]. Consequently, the optimal ultrasonic power was determined to be 250 W.

#### 3.2.3 Effect of extraction time on neuroprotective activity of extracts

Extraction time is a critical parameter in ultrasonic-assisted extraction, significantly influencing the extraction outcomes. Fig. 2C shows, the activity of the extracts showed an increasing trend within the 10 to 20 min extraction window, reaching its peak value of 81.09 ± 1.33 % at 20 min. First, prolonged extraction time enhances the ultrasonication induced cavitation effect, promoting microbubble generation, expansion, and implosion within the solvent. This process generates intense shockwaves that mechanically degrade plant cellular structures [54,55]. Particularly, in RPL tissues, characterized by cellulose-hemicellulose-pectin matrices in cell walls and middle lamellae, the ultrasonic cavitation disrupts these matrices, facilitating the release of bioactive compounds into the extraction medium. Consequently, the activity of the extracts was significantly improved during the early extraction phase [56,57]. Moreover, continuous mechanical vibration evenly destroys cellular matrices, improving bioactive compounds release. However, as the extraction time increases beyond 20 min, the activity of the extracts slightly declines, showing a significant downward trend after 40 min. This may be result from the degradation or deactivation of active components due to prolonged ultrasonic treatment through thermal effects and leaching of impurities [58,59]. The similar observation has been reported for the extraction of bioactive substances from ligustri lucidi fructus and *Acanthopanax senticosus* fruits [42,60]. Therefore, an appropriate extraction time can enhance extraction efficiency while maintaining the stability of active components. In this experiment, an extraction time of 20 min was identified as optimal for further investigation.

#### 3.2.4 Effect of liquid-to-solid ratio on neuroprotective activity of extracts

The liquid-to-solid ratio represents another key factor in the extraction process, significantly affecting both the efficiency of solvent-plant material interactions and the dissolution of active components. As demonstrated in Fig. 2D, the activity of the extracts exhibited a clear dependence on the liquid-to-solid ratio. When increasing the liquid-to-solid ratio from 10 to 40 mL/g, the activity of the extracts significantly increased from 74.57 ± 1.29 % to 81.25 ± 0.85 %. This phenomenon results from that an increased liquid-to-solid ratio establishes a stronger differential solute concentration at the solvent-plant interface, thereby enhancing osmotic pressure and subsequently accelerating the diffusion rate of active components [61]. Moreover, the higher liquid-to-solid ratio expands the interfacial solvent- active constituents contact area while simultaneously reducing solvent viscosity. These combined effects facilitate the diffusion of target compounds and potentiate the cavitation effect [62]. However, no significant improvement in extract activity was observed upon further increasing the liquid-to-solid ratio (*p* > 0.05). This finding aligned with previous reports regarding ultrasound-assisted extraction of triterpenoids from *Astragalus membranaceus* flowers and *Jatropha curcas* leaves, as well as polyphenol extraction from pomegranate seeds [49,63,64]. Through comprehensive evaluation of both extraction efficiency and cost-effectiveness, a liquid-to-solid ratio of 40 mL/g was established as the optimal condition for subsequent experimental investigations in this study.

### 3.3 Response surface analysis and model validation

#### 3.3.1 Response surface analysis

To systematically optimize extraction parameters and elucidate the relationship between critical process variables and the neuroprotective efficacy of the extract, a response surface methodology (RSM)-based approach was implemented. RSM facilitated the design of an experimental matrix and subsequent analysis through comprehensive model fitting and multivariate statistics. This enabled simultaneous evaluation of individual factor effects, including extraction time, liquid-to-solid ratio, temperature, and ultrasonic power, as well as their synergistic interactions on the primary response variable of neuroprotective activity, as quantified by cell viability. Initial single-factor experiments identified three operational levels (high, medium, low) for each parameter. BBD was then implemented to optimize four key variables: extraction time (X_1_), liquid-to-solid ratio (X_2_), temperature (X_3_), and ultrasonic power (X_4_). The 29-run experimental matrix yielded cell viabilities of 77.92–81.76 % (Table 1), with the response surface analyzed using a quadratic polynomial model (Eq3).

$$Y\text{=81.3100+0.2900}{\text{X}}_{\text{1}}\text{+0.3208}{\text{X}}_{\text{2}}\text{+0.2625}{\text{X}}_{\text{3}}-\text{0.3083}{\text{X}}_{\text{4}}\text{+0.4350}{\text{X}}_{\text{1}}{\text{X}}_{\text{2}}+$$

$$\text{0.0925}{\text{X}}_{\text{1}}{\text{X}}_{\text{3}}-\text{0.2075}{\text{X}}_{\text{1}}{\text{X}}_{\text{4}}\text{+0.0225}{\text{X}}_{\text{2}}{\text{X}}_{\text{3}}\text{+0.1800}{\text{X}}_{\text{2}}{\text{X}}_{\text{4}}-\text{0.2125}{\text{X}}_{\text{3}}{\text{X}}_{\text{4}}-$$

(3) $$\text{1.3200}{\text{X}}_{\text{1}}^{\text{2}}-\text{1.0800}{\text{X}}_{\text{2}}^{\text{2}}-\text{1.6900}{\text{X}}_{\text{3}}^{\text{2}}-\text{1.1900}{\text{X}}_{\text{4}}^{\text{2}}$$

ANOVA validated all response surface models, with the outcomes presented in Table 2. The model showed high significance (*F* = 20.77, *p* < 0.0001), confirming high statistical significance for the UADE of neuroprotective components from RPL. Additionally, the lack-of-fit test (*F* = 1.83, *p* = 0.2945) validated the quadratic model adequately fit the experimental data without significant unexplained variation. The model demonstrated excellent goodness-of-fit (*R*^2^ = 0.9541), significantly above the 0.75 benchmark for adequate predictive accuracy in extraction optimization studies [26,63]. With an adjusted *R*^2^ (*R*2_Adj_) of 0.9081, the regression model explained 90.8 % of response variability, indicating excellent agreement with experimental observations. The close agreement between predicted *R*^2^ (0.7700) and adjusted *R*^2^ (0.9081) meets the < 0.2 benchmark for model consistency, demonstrating robust predictive performance for experimental outcomes. The coefficient of variation (*C.V.*% = 0.4446) indicates the data exhibited excellent fitting and reproducibility [28]. Four parameters (X_1_, X_2_, X_3_, X_4_) significantly influenced RPL extraction (p < 0.05). Additionally, the interactions between X variables and the quadratic terms (X_1_X_2_) had statistically significant effects (*p* < 0.05). Based on *F*-value analysis, the factors affecting the extraction process ranked in order of influence as: X_2_ (liquid-to-solid Ratio) > X_4_ (ultrasonic power) > X_1_ (extraction time) > X_3_ (temperature). Among these factors, the solvent-to-solid ratio was primary determinant of extraction efficiency, followed by ultrasonic power.

**Table 2:** ANOVA for quadratic model.

Source	Sum of Squares	df	Mean Square	*F*-value	*p*-value
Model	35.99	14	2.57	20.77	< 0.0001
X_1_	1.01	1	1.01	8.15	0.0127
X_2_	1.24	1	1.24	9.98	0.0070
X_3_	0.8269	1	0.8269	6.68	0.0216
X_4_	1.14	1	1.14	9.22	0.0089
X_1_X_2_	0.7569	1	0.7569	6.12	0.0268
X_1_X_3_	0.0342	1	0.0342	0.2765	0.6072
X_1_X_4_	0.1722	1	0.1722	1.39	0.2578
X_2_X_3_	0.0020	1	0.0020	0.0164	0.9000
X_2_X_4_	0.1296	1	0.1296	1.05	0.3235
X_3_X_4_	0.1806	1	0.1806	1.46	0.2470
X_1_2	11.27	1	11.27	91.06	< 0.0001
X_2_2	7.56	1	7.56	61.06	< 0.0001
X_3_2	18.51	1	18.51	149.58	< 0.0001
X_4_2	9.20	1	9.20	74.30	< 0.0001
Residual	1.73	14	0.1238		
Lack of Fit	1.42	10	0.1422	1.83	0.2945
Pure Error	0.3111	4	0.0778		
Cor Total	37.72	28			
Std. Dev.	0.3518		*R*^2^	0.9541	
Mean	79.12		*R*^2^_Adj_	0.9081	
*C*.*V*.%	0.4446		*R*^2^_Pred_	0.7700	
			Adeq precision	13.7050	

**Fig. 3:**
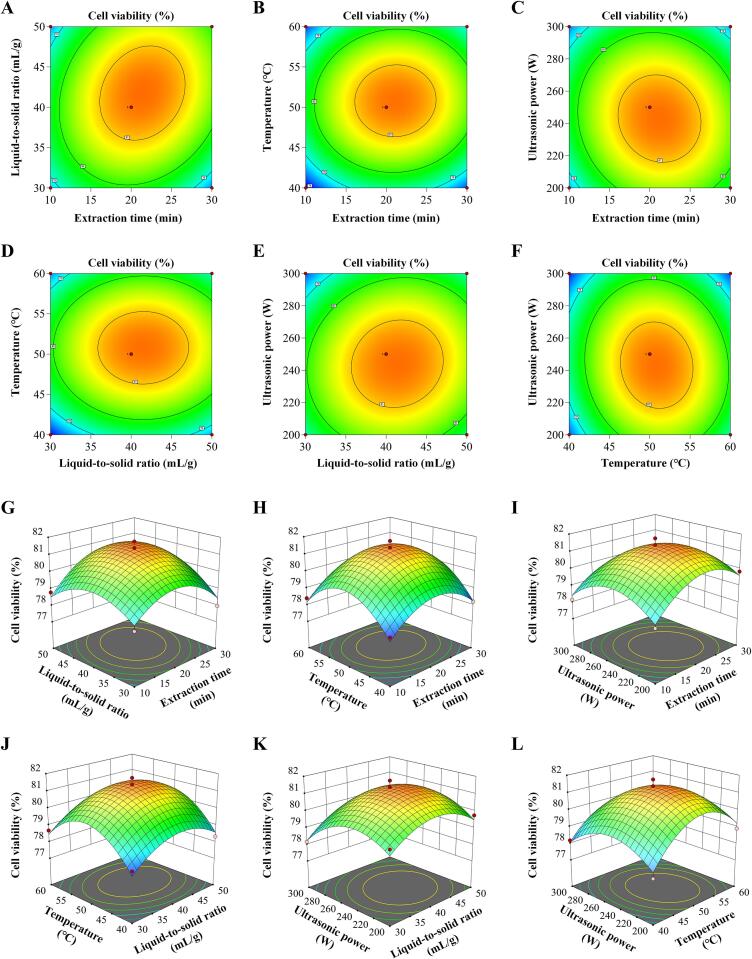
Response surface and contour plots depicting the interactions between the following pairs of variables: X_1_ and X_2_ (A, G), X_1_ and X_3_ (B, H), X_1_ and X_4_ (C, I), X_2_ and X_3_ (D, J), X_2_ and X_4_ (E, K), and X_3_ and X_4_ (F, L) on neuroprotective activity of RPL extracts.

RSM employed 3D surface plots and 2D contour plots to visualize interactions between independent variables and their effects on neuroprotective activity, with other factors held constant. As shown in Fig. 3, the neuroprotective activity of the extracts exhibited significant sensitivity to X_1_, X_2_, X_3_, and X_4_ (*p* < 0.05), consistent with ANOVA results. The pronounced 3D curvature and elliptical 2D contours indicated that the X_1_X_2_ interaction significantly affected neuroprotective activity (*p* < 0.05), demonstrating significant variable interdependence. In contrast, near-circular 2D contours and shallow 3D surfaces indicated negligible other interaction effects on neuroprotection (*p* > 0.05), consistent with ANOVA. Consistent with established literature, the extraction time and liquid-to-solid ratio exhibited significant interactive effects on bioactive compound extraction [58,65]. Through numerical optimization using the desirability function, the optimal conditions for maximum neuroprotective activity were determined to be: extraction time: 21.52 min; liquid-to-solid ratio: 41.68 mL/g; temperature: 50.92 °C; ultrasonic power: 243.09 W.

#### 3.3.2 Model validation and comparison of extraction methods

To validate the predictive model's reliability, we slightly adjusted the extraction conditions to practical settings: extraction time at 22 min; liquid-to-solid ratio at 42 mL/g; temperature at 51 °C; ultrasonic power at 243 W. Under these conditions, the UADE of RPL resulted in a cell viability of 81.44 ± 0.18 %, aligning with predictions (81.39 %), demonstrating model stability.

**Fig. 4:**
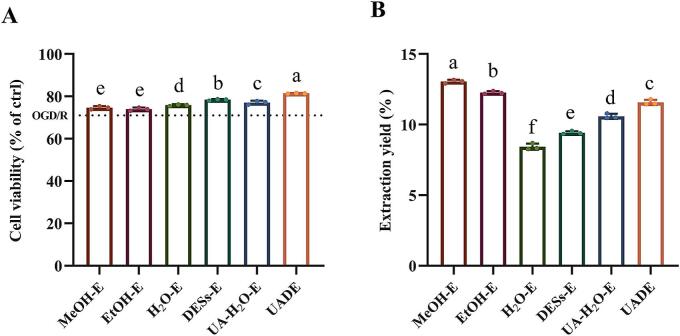
Comparative evaluation of extraction methods for neuroprotective components from RPL. (A) Effects of extraction methods on cell viability of neuroprotective components. (B) Extraction yield of various extraction methods. (Different letters indicate the statistical significance at *p* < 0.05).

Under the aforementioned optimized extraction conditions (extraction time: 22 min; liquid-to-solid ratio: 42 mL/g; temperature: 51 °C; ultrasonic power: 243 W), the H_2_O-E of RPL (cell viability of 75.87 ± 0.43 %) demonstrated superior bioactivity compared to EtOH-E (cell viability of 73.97 ± 0.76 %) and MeOH-E (cell viability of 74.66 ± 0.74 %), while the DESs-E exhibited even greater neuroprotective efficacy (cell viability of 78.42 ± 0.41 %) than these three traditional solvents (Fig. 4A). Ultrasound assistance further enhanced the neuroprotective activity of both UA-H_2_O-E and UADE extracts, with UADE showing the highest potency. These findings aligned with established extraction patterns observed for other bioactive compounds, including phenols, flavonoids, triterpenoids, and curcuminoids, where UADE consistently demonstrated optimal performance [36,50,[65], [66], [67], [68], [69], [70]]. Notably, while ultrasound assistance increased the extraction yields for both UA-H_2_O-E and UADE methods, UADE did not achieve the highest extraction efficiency, which contrasts with its superior neuroprotective activity profile (Fig. 4B). This phenomenon may be attributed to the differential dissolution properties of bioactive constituents and other components in RPL. The extensive hydrogen-bonding network in DESs facilitates enhanced solvent–solute interactions, explaining its superior extraction efficiency relative to conventional solvents [71]. Moreover, the optimized DESs formulation demonstrates remarkable selectivity for target neuroprotective compounds, thereby increasing neuroprotective constituent extraction efficiency and reducing co-extraction of impurities [30,72]. When combined with ultrasound assistance, which significantly further improves neuroprotective compounds dissolution kinetics [73]. Moreover, compared to conventional extraction methods for active compounds in RPL, the UADE approach reduced solvent consumption, shortened extraction time, and improved extraction efficiency [12,17,[74], [75], [76]]. These synergistic improvements established UADE as the optimal methodology for neuroprotective compounds extraction from RPL.

### 3.4 Qualitative and quantitative analysis of chemical compounds

**Fig. 5:**
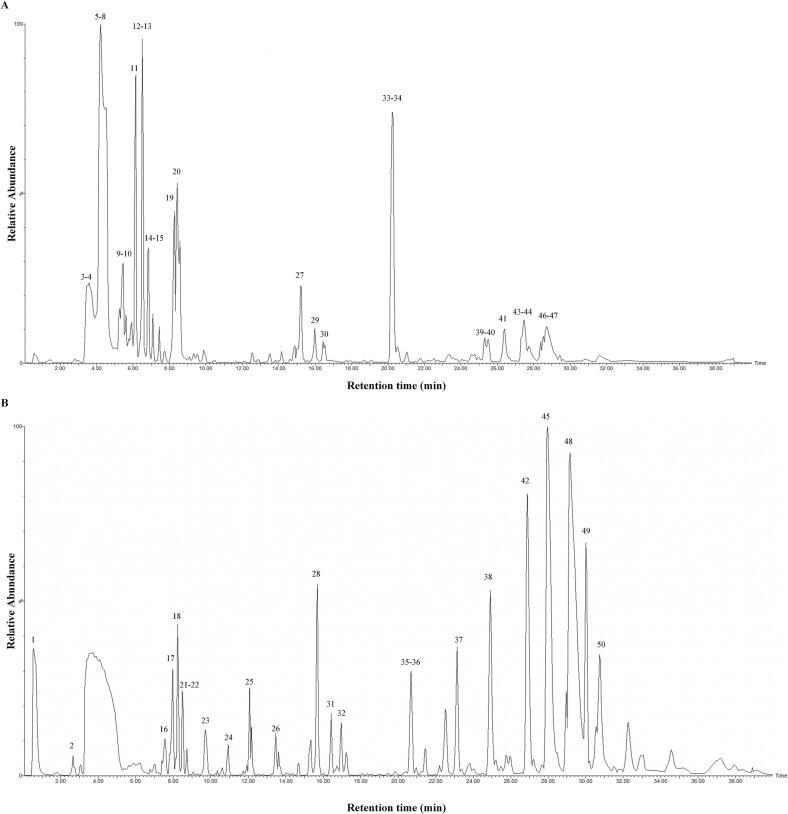
The base peak intensity (BPI) chromatograms of RNPE from UPLC-Q-TOF-MS analysis. (A) Negative scan; (B) Positive scan.

Initially, the neuroprotective active components were purified via AB-8 resin chromatography to yield RNPE. The UPLC-Q-TOF-MS analysis characterized the RNPE composition. RNPE’s base peak intensity (BPI) chromatograms are shown in Fig. 5. Processing of MS data identified 50 compounds, and detailed mass spectral information of these constituents is summarized in Table 3. Phytochemical characterization revealed a diverse array of bioactive constituents in RNPE, including 30 terpenoids (including 12 triterpenes, 5 characteristic monoterpenes, 2 diterpenes, and 1 sesquiterpene), along with 8 phenolics, 7 sterols, 4 alkaloids, 4 aliphatics, 3 flavonoids and so on. Among the identified compounds, paeoniflorin [77], sweroside [78], hyperforin [79], eplerenone [80], crustecdysone [81], allopregnanolone [82], hericenone F [83], desoxyrhaponticin [84], pipernonaline [85], and soyacerebroside I [86] have been demonstrated significant neuroprotective activity. This comprehensive chemical profile demonstrates both the structural diversity of RNPE constituents and their potential to mediate neuroprotective effects through multiple pharmacological mechanisms.

**Table 3:** UPLC parameters for detection of RNPE.

**NO.**	**Rt (min)**	**Identification**	**Formular**	**Ion mode**	**Observed mass (*m*/*z*)**	**Mass error (ppm)**	**Major fragment ion**	**Mode**
**1**	0.575	Norvaline	C_5_H_11_NO_2_	[M + H]^+^	118.0871	−2.721778033	102.0579, 101.0660	+
**2**	2.652	1-Undecene	C_11_H_22_	[M + H]^+^	155.1809	−6.117401093	139.1242, 97.1003	+
**3**	3.542	Paeoniflorin	C_23_H_28_O_11_	[M−H]^-^	479.1537	3.467998342	449.1176, 327.1075, 165.0549, 121.0372	−
**4**	3.554	Ganosporeric acid A	C_30_H_38_O_8_	[M−H]^-^	525.2474	2.795189269	507.2347, 479.2134	−
**5**	4.273	Sweroside	C_16_H_22_O_9_	[M−H]^-^	357.1221	−9.85028552	197.0935, 151.0717, 127.0366, 111.0725	−
**6**	4.324	Mudanpioside D	C_24_H_30_O_12_	[M−H]^-^	509.1610	9.675411776	479.1596, 357.1221, 327.1112, 309.1024, 165.0549	−
**7**	4.324	Ginsenoside B2	C_48_H_82_O_18_	[M−H]^-^	945.547	−4.954024714	843.5151, 509.3136	−
**8**	4.576	(R)-Heraclenol 2′-(3-methyl-2-butenoate)	C_21_H_22_O_7_	[M−H]^-^	385.1291	−0.900893525	341.1378, 201.0123, 185.0247, 173.0229	−
**9**	5.413	Cartormin	C_27_H_29_NO_13_	[M−H]^-^	574.1529	5.555945597	556.1440, 532.1452, 394.0920	−
**10**	5.413	Safflomin C	C_30_H_30_O_14_	[M−H]^-^	613.1608	−8.226898229	595.1401, 405.0900, 119.0547	−
**11**	6.148	Isoswertiajaponin	C_22_H_22_O_11_	[M−H]^-^	461.1084	0.024961592	446.0873, 418.0934	−
**12**	6.501	Mudanpioside E	C_24_H_30_O_13_	[M−H]^-^	525.1628	−3.730266705	345.1614, 327.1038, 311.6113	−
**13**	6.602	1-O-Galloylfructose	C_13_H_16_O_10_	[M−H]^-^	331.0636	8.90062747	303.0724, 289.0580, 275.0797	−
**14**	6.754	Epoxygedunin	C_28_H_34_O_8_	[M−H]^-^	497.2193	−3.483304113	461.1876, 91.0590	−
**15**	6.855	Lariciresinol-sesquilignan	C_30_H_36_O_10_	[M−H]^-^	555.2214	2.966987786	525.2192, 511.1928, 207.2024	−
**16**	7.539	Hyperforin	C_35_H_52_O_4_	[M + H]^+^	537.3976	−6.028831414	497.3208, 481.3333	+
**17**	7.971	Pipernonaline	C_21_H_27_NO_3_	[M + H]^+^	342.2069	−0.018351471	312.1880, 257.1135	+
**18**	8.224	(3E,9E,12E)-Hexadeca-3,9,12-trienedioylcarnitine	C_23_H_37_NO_6_	[M + H]^+^	424.2669	7.042451244	365.1907, 85.0291	+
**19**	8.274	7-Methyl-1,4,5-naphthalenetriol 4-[xylosyl-(1->6)-glucoside]	C_22_H_28_O_12_	[M−H]^-^	483.1510	−1.497836254	465.1433, 441.1413, 425.1451	−
**20**	8.426	4,7-Didehydroneophysalin B	C_28_H_28_O_9_	[M−H]^-^	507.1653	0.458371055	377.0607, 361.0088	−
**21**	8.476	Quercetin 7,3′,4′-trimethyl ether	C_18_H_16_O_7_	[M + H]^+^	345.0973	0.298031799	330.0741, 329.0265	+
**22**	8.476	Neoporrigenin B	C_27_H_42_O_5_	[M + H]^+^	447.3086	5.420031851	431.2714, 417.3031, 331.2239	+
**23**	9.717	Desoxyrhaponticin	C_21_H_24_O_8_	[M + H]^+^	405.1532	4.239662225	317.1320, 289.1429	+
**24**	10.934	Melilotussaponin O2	C_47_H_72_O_19_	[M + H]^+^	941.4769	−2.46415575	855.4747, 667.3688	+
**25**	12.074	4-methylpentadecanoic acid	C_16_H_32_O_2_	[M + NH_4_]^+^	274.2752	−4.264862404	95.0883, 83.0891	+
**26**	13.465	Eplerenone	C_24_H_30_O_6_	[M + H]^+^	415.2148	−6.650409292	397.2060, 195.1073	+
**27**	15.188	Lucidenic acid G	C_27_H_40_O_7_	[M + e]^-^	476.2798	−3.981687972	429.2661, 415.2323, 399.2547	−
**28**	15.642	Epicatechin-(2beta->7,4beta->6)-catechin	C_30_H_24_O_12_	[M + H]^+^	577.1380	−5.932411852	531.1022, 519.1261, 501.0836, 589.0736	+
**29**	15.974	Crustecdysone	C_27_H_44_O_7_	[M + e]^-^	480.3131	−8.111982801	478.2943, 433.2449, 345.1919	−
**30**	16.428	Contignasterol	C_29_H_48_O_7_	[M−H]^-^	507.3362	−7.876752096	589.3232, 477.3313, 475.3009	−
**31**	16.428	2-Polyprenyl-6-methoxy-1,4-benzoquinone	C_17_H_22_O_3_	[M + H]^+^	275.1668	−7.631792886	257.1561, 247.1645	+
**32**	16.961	Ganoderic acid eta	C_30_H_44_O_8_	[M + H]^+^	533.3068	8.659780835	501.2852, 497.2934, 471.3116	+
**33**	20.227	Ophiopogonin C	C_39_H_62_O_12_	[M + e]^-^	722.4276	−4.114740676	575.3467, 485.3244, 177.0406	−
**34**	20.227	9alpha-(3-Methyl-2E-butenoyloxy)-4S-hydroxy-10(14)-oplopen-3-one 4-acetate	C_22_H_32_O_5_	[M−H]^-^	375.2137	9.258957851	251.1669, 233.1597	−
**35**	20.682	Allopregnanolone	C_21_H_34_O_2_	[M + NH_4_]^+^	336.2869	8.267642936	299.2049, 289.2194	+
**36**	20.682	Ethyl linolenate	C_20_H_34_O_2_	[M + H]^+^	307.2609	9.048654879	261.2281, 147.1164, 133.1036	+
**37**	+ 23.140	1-Stearoylglycerol	C_21_H_42_O_4_	[M + H]^+^	359.3172	−3.034041531	341.3102, 267.2721	+
**38**	24.91	Hericenone F	C_35_H_54_O_6_	[M + H]^+^	571.3957	7.244384255	551.3723, 317.1757	+
**39**	25.317	Ginsenoside Rh8	C_36_H_60_O_9_	[M−H]^-^	635.4109	7.921567173	547.4030, 477.3940	−
**40**	25.317	Az I	C_48_H_74_O_18_	[M−H]^-^	937.4738	6.310013862	895.4732, 893.4937, 877.4997	−
**41**	26.355	Neogitogenin	C_27_H_44_O_4_	[M−H]^-^	431.3134	6.398740082	415.2824, 413.3096	−
**42**	26.888	Soyacerebroside I	C_40_H_75_NO_9_	[M + H]^+^	714.5524	−0.583624439	696.5548, 678.5319, 670.5280	+
**43**	27.444	Cynarasaponin H	C_47_H_74_O_18_	[M + e]^-^	926.4951	−7.646607275	851.4729, 835.4407, 795.5007	−
**44**	27.444	26-desgluco-avenacoside A	C_45_H_72_O_18_	[M−H]^-^	899.4702	−6.820239113	881.4435, 869.4390, 825.4316	−
**45**	27.977	Soyasaponin IV	C_41_H_66_O_13_	[M + H]^+^	767.4620	−5.026775779	749.4341, 707.4526, 665.4243	+
**46**	28.712	Torvoside F	C_45_H_74_O_18_	[M−H]^-^	901.4803	−0.648367334	899.5002, 859.4620, 829.4516	−
**47**	28.864	2-Deoxy-25-Methyldolichosterone	C_29_H_48_O_4_	[M−H]^-^	459.3432	9.36110461	457.3323, 443.3174	−
**48**	29.167	CE(3 M5)	C_40_H_64_O_3_	[M + H]^+^	593.4949	−2.618563478	577.4625, 523.4150	+
**49**	30.003	Sanguiin H3	C_68_H_48_O_44_	[M + H]^+^	1569.1463	8.504424674	1551.1498, 1539.1560, 465.0240	+
**50**	30.738	Camelliin A	C_68_H_48_O_44_	[M + H]^+^	1569.1505	5.82783277	1551.1422, 1399.1431	+

### 3.5 *In vivo* neuroprotective effects of RNPE

**Fig. 6:**
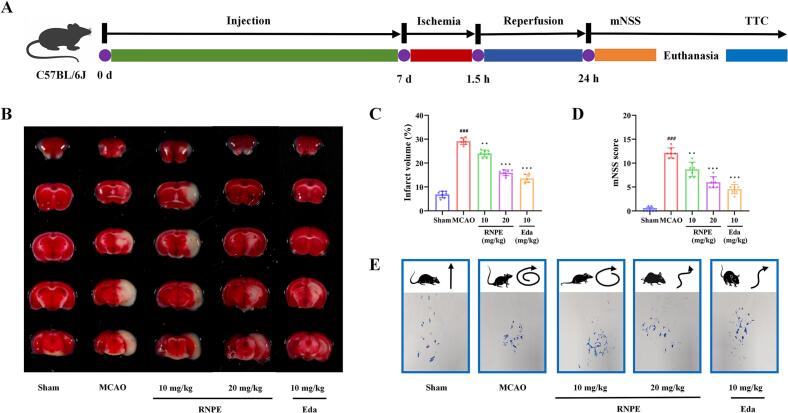
RNRE alleviates MCAO mice brain injury and neurological deficits (n = 7). (A) The schematic illustration of the *in vivo* experiment process. (B) Representative images of TTC staining of brain tissue, the red and white colour represent the normal and infarcted area, respectively. (B) Treatment with RNPE significantly reduced the brain infarction area. (D) mNSS score. (E) Gait trajectory testing. (n = 7, #compared with the sham group, ###*p* < 0.001. *compared with the MCAO group, *p < 0.05; **p < 0.01; ***p < 0.001). (For interpretation of the references to colour in this figure legend, the reader is referred to the web version of this article.)

To evaluate the neuroprotective potential of the RNPE, an MCAO model assessed *in vivo* efficacy. This model is widely used to simulate stroke-like conditions and assess neuroprotective agents. MCAO modeling in mice typically triggers hemiplegia, ischemic injury, infarction, body weight reduction, and lasting neurological dysfunction [87]. In this study, TTC staining was performed to quantify RNPE's effects on MCAO-induced cerebral infarction. The brain infarction area in MCAO mice without any intervention was 26.7 %. RNPE treatment reduced cerebral infarction area to 17.7 % (Fig. 6B and 6C). Neurological function was evaluated using the mNSS. The MCAO mice exhibited significant neurological impairment, while the RNPE treated group demonstrated improved scores, indicating functional recovery (Fig. 6D). The MCAO mice gait trajectory testing revealed hemiplegia, demonstrated by rotation toward the paretic side and impaired linear locomotion, whereas RNPE treatment significantly alleviated these hemiplegic symptoms (Fig. 6E). These results demonstrate that RNPE possesses significant neuroprotective effects in MCAO mice.

The close structure–activity relationship exists between the observed neuroprotective effects of RNPE and identified chemical constituents [88]. Terpenoids constitute the primary bioactive components in RNPE, with both monoterpenes and diterpenes demonstrating well-documented neuroprotective effects. Notably, compound 3 (paeoniflorin), the most extensively studied monoterpene glycoside, demonstrates multi-target mechanisms against CIRI [18]. Recent studies reveal that the combination of paeoniflorin with atractylenolide I and atractylenolide III enhances angiogenesis and neural recovery in ischemic stroke mouse models by promoting cellular proliferation/migration and upregulating angiogenic proteins (e.g., IGF-2) [89]. This compound further potentiates limb remote ischemic postconditioning by modulating neutrophil NADPH pathway to alleviate CIRI [90]. In addition, monoterpene glycosides compounds 5 and 16 also showed neuroprotective effects. Compound 5 (sweroside) achieves neuroprotective effects by decreasing AChE activity and MDA level along with upregulating of bdnf, npy, egr1, nfr2a, and creb1 gene expression [91]. Compound 16 (hyperforin) promotes neurovascular regeneration and functional recovery after stroke through IL-6-mediated negative immune regulation [92]. Deeper mechanistic studies reveal that compound 16 exerts antidepressant activity through selective activation of TRPC6 [93]. Among other terpenoids, the sesquiterpene compound 34 displays marked anti-neuroinflammatory activity by significantly inhibiting LPS-stimulated NO release in BV-2 microglia [94]. Compound 35 (allopregnanolone, an FDA-approved neurosteroid for postpartum depression), mitigates blood–brain barrier (BBB) damage following CIRI through GABAA receptor modulation and concurrently promotes hippocampal neurogenesis [95]. Phenolic compound 23 (desoxyrhaponticin) inhibits the aggregation of BACE1 and Aβ, thereby exerting a neuroprotective effect in Alzheimer’s disease [84]. Although the 12 identified triterpenes (the most abundant chemical class in RNPE) lack direct neuroprotection studies, their structural analogs show significant CIRI mitigation, suggesting analogous bioactivity [[96], [97], [98], [99], [100]]. Collectively, these findings validate UADE as an effective method for extracting neuroprotective components from RPL.

## 4 Conclusion

This study establishes the first systematic UADE protocol for isolating neuroprotective components from RPL, combining activity-guided screening with *in vitro* disease models to identify the optimal DESs system and optimize RSM parameters. Comparative analysis demonstrated the superior efficacy of UADE in enhancing RPL extract neuroprotection compared to conventional heat and standard DESs extraction methods. Following AB-8 macroporous resin purification, UPLC-Q-TOF-MS confirmed neuroprotective compounds in the RNPE, with *in vivo* MCAO mice experiments validating their therapeutic potential. This study integrates green extraction technologies with systematic pharmacological validation to establish an effective strategy for demonstrating RPL’s efficacy against ischemic stroke and other neurological diseases for the first time. This work provides both theoretical basis for bioactive compound extraction and practical applications for UADE technology.

## CRediT authorship contribution statement

**Yongkang Xue:** Writing – original draft, Methodology, Investigation, Data curation. **Zhengyu Hu:** Writing – review & editing, Supervision, Conceptualization. **Yuxin Jiang:** Methodology, Investigation. **Man Li:** Methodology, Investigation. **Yanan Liu:** Software, Methodology. **Yuanqi Duan:** Software. **Wei Zhou:** Supervision, Resources, Project administration. **Jinfeng Sun:** Writing – review & editing, Project administration, Methodology. **Gao Li:** Project administration, Conceptualization.

## Declaration of competing interest

The authors declare that they have no known competing financial interests or personal relationships that could have appeared to influence the work reported in this paper.
