# Supplementary material for: Ultrasound-assisted deep eutectic solvent of neuroprotective extracts from Paeonia lactiflora Pall. root: Process optimization, compositions characterization, and neuroprotective activity validation
Source: Ultrason Sonochem. 2025 Jun 27;120:107449. doi: 10.1016/j.ultsonch.2025.107449 (PMC12270807; doi:10.1016/j.ultsonch.2025.107449)
Supplement: Supplementary Data 1 [file mmc1.docx]

# Supplementary Material

**Table S1. DESs components.**

| **Name*** | **Combination** | **Mole ratio** |
| --- | --- | --- |
| PDES | L-Proline : Glycerol | 1:3 |
| HDES | Betaine monohydrate : Benzyl alcohol | 1:3 |

*****HDES: hydrophobic deep eutectic solvent, PDES: hydrophilic deep eutectic solvent.

**Table S2. Factors and levels for Box Behnken design.**

| **Independent variables** | **Label** | **Levels** | | |
| --- | --- | --- | --- | --- |
|  |  | -1 | 0 | 1 |
| Extraction time (min) | X_1_ | 10 | 20 | 30 |
| Liquid-to-solid ratio (mL/g) | X_2_ | 30 | 40 | 50 |
| Temperature (°C) | X_3_ | 40 | 50 | 60 |
| Ultrasonic power (W) | X_4_ | 200 | 250 | 300 |

**Table S3. UPLC parameters for detection of RNPE.**

| **Positive ion** | | | **Negative ion** | | |
| --- | --- | --- | --- | --- | --- |
| Time (min) | A% | B% | Time (min) | A% | B% |
| 0 | 95 | 5 | 0 | 95 | 5 |
| 2 | 95 | 5 | 2 | 95 | 5 |
| 30 | 2 | 98 | 30 | 2 | 98 |
| 35 | 2 | 98 | 35 | 2 | 98 |
| 39 | 95 | 5 | 39 | 95 | 5 |
| 40 | 95 | 5 | 40 | 95 | 5 |

**
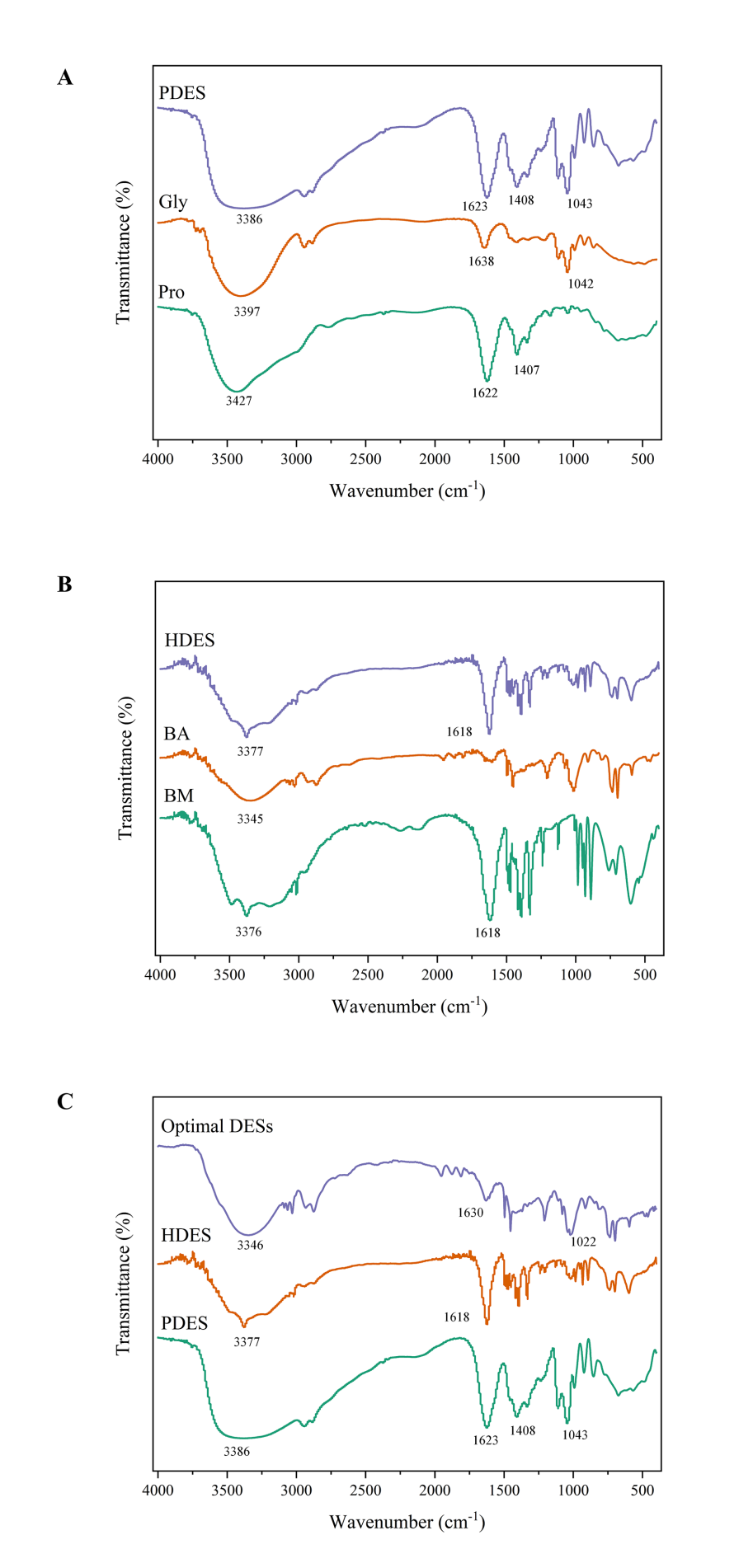
Fig. S1** FTIR spectra of DESs. (A) PDES. (B) HDES. (C) Optimal DESs.
